# Analyzing Blood Cells of High-Risk Myelodysplastic Syndrome Patients Using Interferometric Phase Microscopy and Fluorescent Flow Cytometry

**DOI:** 10.3390/bioengineering11030256

**Published:** 2024-03-06

**Authors:** Itay Barnea, Lior Luria, Arik Girsault, Ofira Dabah, Matan Dudaie, Simcha K. Mirsky, Drorit Merkel, Natan T. Shaked

**Affiliations:** 1 Department of Biomedical Engineering, Tel Aviv University, Tel Aviv 6997801, Israel; iiorluria@mail.tau.ac.il (L.L.); arikgirsault@tauex.tau.ac.il (A.G.); ofiradabahyo@tauex.tau.ac.il (O.D.); matan.dudaie@gmail.com (M.D.); simchamirsky@mail.tau.ac.il (S.K.M.); nshaked@tauex.tau.ac.il (N.T.S.); 2 MDS Center, Sheba Medical Center, Ramat Gan 5266202, Israel; drorit.merkel@sheba.health.gov.il; 3 Faculty of Medicine, Tel Aviv University, Tel Aviv 6997801, Israel

**Keywords:** myelodysplastic syndromes, interferometric phase microscopy, hematology

## Abstract

Myelodysplastic syndromes (MDSs) are a group of potentially deadly diseases that affect the morphology and function of neutrophils. Rapid diagnosis of MDS is crucial for the initiation of treatment that can vastly improve disease outcome. In this work, we present a new approach for detecting morphological differences between neutrophils isolated from blood samples of high-risk MDS patients and blood bank donors (BBDs). Using fluorescent flow cytometry, neutrophils were stained with 2′,7′-dichlorofluorescin diacetate (DCF), which reacts with reactive oxygen species (ROS), and Hoechst, which binds to DNA. We observed that BBDs possessed two cell clusters (designated H and L), whereas MDS patients possessed a single cluster (L). Later, we used FACS to sort the H and the L cells and used interferometric phase microscopy (IPM) to image the cells without utilizing cell staining. IPM images showed that H cells are characterized by low optical path delay (OPD) in the nucleus relative to the cytoplasm, especially in cell vesicles containing ROS, whereas L cells are characterized by low OPD in the cytoplasm relative to the nucleus and no ROS-containing vesicles. Moreover, L cells present a higher average OPD and dry mass compared to H cells. When examining neutrophils from MDS patients and BBDs by IPM during flow, we identified ~20% of cells as H cells in BBDs in contrast to ~4% in MDS patients. These results indicate that IPM can be utilized for the diagnosis of complex hematological pathologies such as MDS.

## 1. Introduction

Myelodysplastic syndromes (MDSs), also known as myelodysplastic neoplasms, are a group of hematopoietic malignancies characterized by marrow dysplasia, with failure of bone marrow stem cells to mature into normal-functioning blood cells. Patients diagnosed with MDS present with anemia, neutropenia, and thrombocytopenia along with morphologic dysplastic bone marrow and an increased risk of acute myeloid leukemia (AML) [1]. Often, these patients suffer from fatigue, recurrent infections, and bleeding. 

The incidence of new MDS diagnoses in the United States is about 4 in 100 thousand per year [1], and the 5-year life expectancy of MDS patients is about 37% [2]. MDS affects mostly elderly patients, with a median age of diagnosis of approximately 70 years, thus the incidence rate increases from 4 per thousand to 25 per 100 thousand above the age of 65. MDSs are broadly divided into those which present a high and low risk of transforming into AML. High-risk patients are typically characterized by worse anemia, neutropenia, or thrombocytopenia and a higher percentage of myoblasts, as well as genetic variants associated with a worse prognosis [3].

The clinical manifestation of MDS is influenced by the specific functional deficiencies present in the particular blood cells. For example, MDS patients with maturational defects of megakaryocytes typically present with bleeding, independently of thrombocytopenia [4]. Similarly, infections are a major cause of illness and death in MDS patients [5], both in patients with neutrophil dysfunction and in patients with neutropenia [6]. Morphological and physiological abnormalities are common in neutrophils of MDS patients, including nuclear hyper-segmentation, cytoplasmic hypo-granularity [7], and a reduction in reactive oxygen species (ROS), which are essential for the management of pathogens [8].

The current protocol for the diagnosis of MDS includes bone marrow aspiration after anemia thrombocytopenia or neutropenia that persists for 6 months or more [2]; thus, even in the most favorable circumstances, patients will receive a diagnosis and start treatment many months after the onset of symptoms. Unfortunately, in some cases, rapid diagnosis and treatment are critical to prevent deterioration in the health of patients. For example, some MDS patients are at high risk of developing AML. These patients may benefit from earlier bone marrow transplantation that may prevent this deadly disease [9]. 

2′,7′-dichlorodihydrofluorescein diacetate, also known as DCF, is a cell-permeable molecule that becomes fluorescent in the presence of ROS [10]. DCF has been used to characterize the presence and distribution of ROS in different living cells including neutrophils. In neutrophils, the level of DCF fluorescence is correlated to the initiation of an immune response to a microorganism stimulation [11,12].

Interferometric phase microscopy (IPM), also known as digital holographic microscopy or quantitative phase microscopy, is a stain-free imaging technique that captures both the amplitude and phase profiles of the light that passes through transparent biological cells. IPM provides a quantitative measurement of the optical path delay (OPD) at each point in the sample, where OPD is defined as the difference between the integral refractive index of the sample and that of the surrounding medium multiplied by the sample thickness. These quantitative quasi-3D phase images enable the calculation of 3D parameters such as the cellular dry mass, in addition to 2D parameters such as cell area [13,14]. 

IPM technologies have been used to distinguish different types of cells by our group and others, for example, to distinguish between normal and abnormal sperm cells [15], between different leucocytes [16] including T cells of different activation modes [17], and between normal and pathological hematopoietic cells such as acute myeloid leukemia (AML) and myeloproliferative neoplasm (MPN) [18]. IPM combined with flow cytometry was previously used for the classification of different types of leucocytes [17,18].

In this work, we present a new way to detect MDS by imaging neutrophils through IPM. This study aims to show that in healthy donors, two neutrophil populations are present in the OPD images, whereas in MDS patients, only one population is present, thereby enabling MDS detection.

## 2. Materials and Methods

### 2.1. Blood Acquisition

High-risk MDS patient blood was acquired from the Hemato-oncology Department of Sheba Medical Center, Ramat Gan, Israel. This study was approved by the Sheba Medical Center (approval number 8374-21-SMC) and the Tel Aviv University Ethical Review Board (approval number 003618-3). MDS patients signed informed consent forms before participating in the study, and blood tubes were coded by numbers in order to preserve the anonymity of the donors. Venous blood was drawn from MDS patients during regular follow-up treatment and placed in tubes containing EDTA. 

As a control group, EDTA-supplemented blood samples from healthy blood bank donors (BBDs) were acquired from the Israeli blood bank, Magen David Adom, Tel Hashomer Hospital, Israel. In all cases, the blood was stored at 4 °C in tubes containing EDTA and tested within 24 h of being drawn.

### 2.2. Neutrophil Isolation

Neutrophils were isolated using the EasySep Direct Human Neutrophil Isolation Kit (StemCell technologies, #19666, Vancouver, BC, Canada). In short, 0.5 mL of blood was placed in round-bottom polystyrene 1.5 mL tubes, where the blood was combined with 50 μL of isolation cocktail and 50 μL of magnetic beads. The mixture was then incubated at room temperature for 5 min, and combined with 3.5 mL of PBS supplemented with 1 mM EDTA. After incubation, the tube was placed in a magnet (EasySep, #18000, Vancouver, BC, Canada) for 5 min, and the liquid was poured into a new tube. Next, 20 μL of isolation cocktail and 50 μL of magnetic beads were added to the tube, and after 5 min of incubation, the tube was placed in the magnet for 5 min, the liquid was then poured into a new tube and centrifuged at 1250 RPM; then, the supernatant was discarded and the cells were resuspended in PBS-EDTA. Cells used for flow cytometry were resuspended in a fluorescent dye mix (see Section 2.3—Flow Cytometry); cells used for imaging in IPM were resuspended in PBS-EDTA supplemented with 2% paraformaldehyde (PFA) fixative, which was required to prevent morphological changes in cells such as degranulation, and with 7 µg/mL Hoechst 33342 for nucleus staining. 

### 2.3. Flow Cytometry

Characterization of cells was performed by two methods of flow cytometry: analytical flow cytometry and flow cytometry that includes cell sorting. In both cases, cells were first stained with 7 µg/mL Hoechst 33342 (Sigma-Aldrich #B2261, Rehovot, Israel) for DNA staining, 2.5 µg/mL 2′,7′-Dichlorofluorescein diacetate (DCF) (Sigma-Aldrich, #D6883) for ROS staining, and 5 µL/mL PE-conjugated anti-human CD66b Antibody (305106, Biolegend, San Diego, CA, USA) in PBS supplemented with 1 mM EDTA for the staining of neutrophils. The cells were incubated with the staining solution at room temperature for 30 min before analysis. 

For analytical flow cytometry, we used the Cytoflex 5L (Beckman Coulter, Brea, CA, USA) system, while for fluorescence-activated cell sorting (FACS), the BD FACSAria III (BD Biosciences, Allschwil, Switzerland) system was used. 

In flow cytometry with sorting, the sorted cells were collected into a 15 mL tube containing paraformaldehyde (PFA). The final concentration of PFA in the sorted cells was ~1%. After sorting, the cells were spun at 1000 RPM, placed in a glass chamber, and imaged by IPM, brightfield microscopy, and fluorescent microscopy. Analysis was performed by the Kaluza analysis software 2.2 (Beckman Coulter).

### 2.4. Optical System

**Figure 1:**
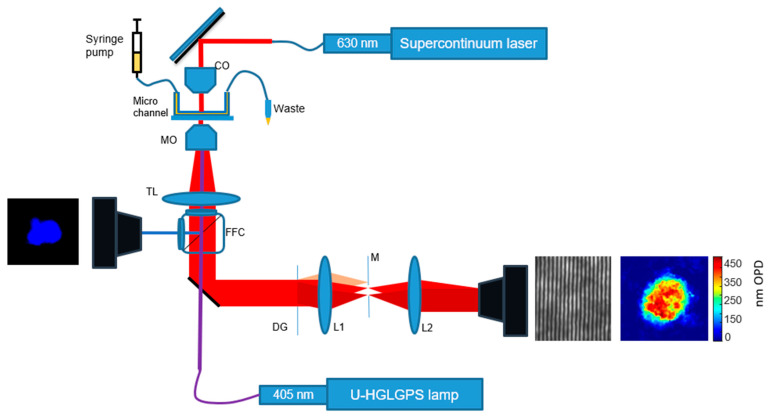
System configuration. The system is based on an Olympus IX83 inverted microscope. Cells stained with Hoechst 33342 are pumped into a microchannel. The cell sample is illuminated by a supercontinuum laser source coupled to an acousto-optic tunable filter. The cells are magnified by a 60× oil microscope objective lens and an interferogram is acquired by the LC-SICA module. In addition, cells are simultaneously imaged by fluorescent or brightfield microscopy by a different camera. CO: condenser; MO: microscope objective lens; TL: tube lens; FFC: fluorescent filter cube; L1 and L2: lenses; DG: diffraction grating; M: mask.

The optical system is shown in Figure 1. It is designed for cells to flow through the optical system in a controlled manner and to capture images of the cells using IPM by low-coherence shearing interferometry, as described in our earlier works [19,20]. The system also contains fluorescent microscopy and brightfield microscopy based on the Olympus IX83 inverted microscope.

For capturing IPM images, we used a low-coherence shearing interferometry with a constant off-axis angle (LC-SICA) module. A supercontinuum laser (SuperK EXTREME, NKT) beam with wavelength of 630 nm and bandwidth of 6 nm illuminates the sample as a plane wave. The sample is imaged with a microscope objective lens (MO) (Olympus PlanApo N 60×/1.42 oil) and a tube lens TL (focal length 200 mm), and the image beam then passes to the external LC-SICA module. As shown in Figure 1, the LC-SICA module consists of a diffraction grating (DG) with 100 lines/mm, generating two laterally shifted sample beams. The diffracted beams are optically Fourier-transformed by lens L1 (focal length 150 mm). Mask M is placed at the conjugate Fourier plane to select only the zeroth and first diffraction orders, and a glass plate phase compensator is positioned in the path of the first order. Then, the two orders are projected onto lens L2 (focal length 300 mm), with L1 and L2 arranged in a 4f imaging configuration. Finally, both beams overlap on the sensor plane of the camera (Thorlabs DCC1545M-GL, 8-bit monochromatic CMOS, 1280 × 1024 pixels of 5.2 µm), creating the off-axis interferogram, captured as an 8-bit BMP image. 

The complex wavefront image was reconstructed from the off-axis interferogram, and the phase of the complex wavefront was extracted and converted to the sample OPD map, where the OPD at a given point (${OPD}_{\left(x,y\right)}$) is defined as the product of the difference between the refractive index of the object $n_{o\left(x,y\right)}$ to that of the surrounding medium $n_{m}$ and the thickness of the object at that point ($h_{o(x,y)}$):

$${OPD}_{\left(x,y\right)}=\left(n_{o\left(x,y\right)}-n_{m}\right)\times h_{o\left(x,y\right)}.$$

The process for the conversion of interferograms to OPD maps is described in [21].

In the resulting OPD maps, the cell area was isolated by a simple threshold, followed by a morphological dilation. Thus, we created a dataset containing the OPD information across the cell areas only. From these maps, we calculated different 3D morphological parameters such as dry mass and the average OPD, and from the threshold maps, we also calculated 2D morphological parameters such as the cell area and the cell perimeter. These IPM-based morphological features are based on our previous works [14,16]. The average OPD (〈OPD〉) is the sum of all OPD values of a given cell divided by the number of pixels in the cell’s projection area. The cellular dry mass (M) is defined as follows:

$$M=\frac{S_{C}}{\alpha }\times \langle OPD\rangle ,$$

where α is the refractive increment, approximated as 0.19 µm^3^/pg, and $S_{C}$ is the projected cell area on the image plane.

In addition to IPM imaging, the Olympus IX83 was also used to capture fluorescent images of the sample simultaneously to the IPM imaging. The light source used for fluorescent imaging was a 130 W mercury lamp (U-HGLGPS). The beam passes a fluorescent filter cube (FFC), illuminates the sample through the microscope objective lens, and then the fluorescent light returns to the FFC and is projected onto the microscope camera (Basler acA2440-75um, Edmund optics, Barrington, NJ, USA). The FFC was set to match the fluorophore used: for Hoechst, an FCC with an excitation filter of 325–375 nm and an emission filter of 435–485 nm was used; for DCF, an FCC with an excitation filter of 450–490 nm and an emission filter of 500–540 nm was used. 

In order to image immobile cells, a glass chamber was built from a #1 60 × 22 mm coverslip, covered by a #1 22 × 22 coverslip. The two coverslips were separated by two 22 mm long sections of a #1 coverslip. The chamber components were bonded together by melted candle wax, creating an empty chamber with a glass floor and roof with open entrances on two opposing sides and a height approximately equal to the thickness of a single #1 coverslip. After the addition of the liquid containing the cells, the chamber entrances on the sides were sealed by melted candle wax to prevent leakage and evaporation. 

To image flowing cells, the cells were pumped by a two-channel programmable syringe pump (NE-4000, New Era Pump Systems, Farmingdale, NY, USA) through a microchannel with a height of 0.1 mm (µ-Slide VI 0.1, Ibidi, Gräfelfing, Germany). In order to identify the flowing cells, in parallel to imaging the cells with IPM, the cells were simultaneously imaged by the fluorescent microscope using the FFC for the Hoechst stain.

### 2.5. Statistical Analysis

Morphologic characteristics of cells captured by IPM were extracted from the OPD maps, quantified, and listed in a table. The statistical significance of the difference between two populations of cells for each characteristic was determined by two-sample *t*-test, while the statistical significance for the difference of proportion between two populations was determined by the two-sample independent proportions test. Results were considered statistically significant for values of *p* < 0.05. Statistical images were generated using Graphpad Prism 9 (GraphPad Software, San Diego, CA, USA).

## 3. Results

### 3.1. Analytical Flow Cytometry

**Figure 2:**
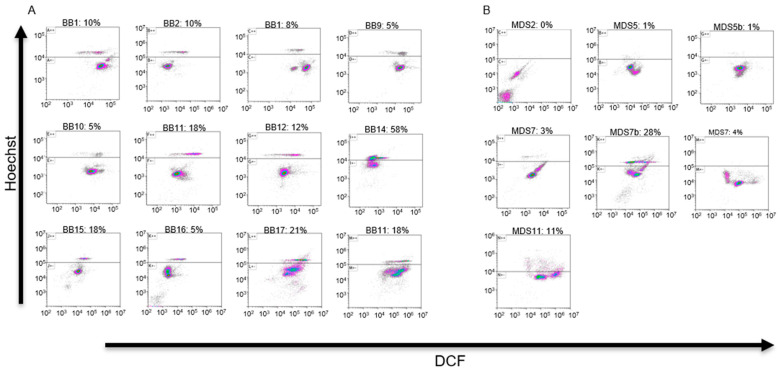
DCF/Hoechst scatter plots of neutrophils isolated from healthy BBDs (**A**) and MDS patients (**B**). Cells are presented after gating to CD66b-positive cells (using PE-αCD66b antibody) or to granulocytes using FSC vs. SSC dispersion scatter plot. Results indicate that healthy BBDs present two distinct subpopulations of cells, separated by the Hoechst level. The percentage of H cells (cells above the threshold line) are presented in each plot. The scatter plots are colored by a gradient of colors (red, green, blue, purple and grey) from highest to the lowest cell density. The average percentage of H cells in the BBD group is 16% compared with 4% in the MDS patient group. The difference between the two groups is statistically significant (*p* < 0.05).

Neutrophils from blood samples of high-risk MDS patients and healthy BBDs were stained with Hoechst, DCF, and PE-CD66b. Cells were analyzed either by the BD FACSAria III or by the Cytoflex 5L systems. Cells are presented on DCF vs. Hoechst scatter plot charts (Figure 2A,B). When the PE-αCD66b conjugate was used, cells were gated to CD66b-positive cells; otherwise, the cells were gated by the FSC vs. SSC scatter plot to include only granulocytes. In all BBD plots, two distinct clusters of cells appear: a cluster with high Hoechst expression and a cluster with low Hoechst expression (see Figure 2A), designated as H and L cells, respectively. The threshold for the distinction between H and L cells (explained in “Cell Sorting”) was 10^4^ for the FACSAria III and 10^5^ for the Cytoflex 5L system, respectively, on the Hoechst axis. The arbitrary fluorescent values of two machines can be calibrated with one another by comparing the ratio of Hoechst fluorescence in the H to L cells analyzed in the different machines. 

The difference between the percentage of H cells in the two groups (18.5% in BBDs vs. 3.4% in MDS patients) is statistically significant (*p* < 0.01). Moreover, scatter plot results indicate that in the MDS group, there is no distinct subpopulation of cells above the threshold (except for the case of MDS7b). Interestingly, the same patient, MDS-7, was tested twice, wherein the second time (MDS7b), blood was drawn shortly after the patient underwent a blood transfusion. In this second sample, a high percentage of H cells was detected, leading us to speculate that these cells were exogenous to the patient. 

### 3.2. Cell Sorting

**Figure 3:**
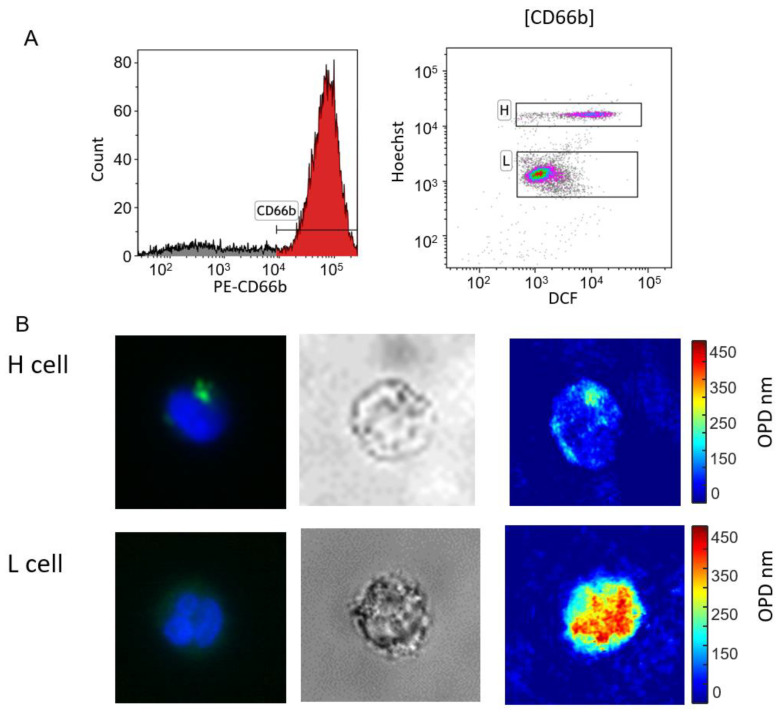
Sorting of high-Hoechst (H) and low-Hoechst (L) cells. (**A**) Neutrophils isolated from a healthy donor (BBD-11) were gated by CD66b expression (colored in red). The gated cells are displayed on a DCF vs. Hoechst scatter plot and colored by a gradient of colors (red, green, blue, purple and grey from high to low cell density). The two subpopulations of cells, H and L cells, were sorted for future analysis by the level of fluorescence and were isolated and fixated. (**B**) A representative cell from each subpopulation, as imaged by fluorescent microscopy with Hoechst (blue) and DCF (green) stains, brightfield, and IPM. Results indicate H cells are characterized by a round nucleus, focal DCF spots, and low OPD in the nucleus. In comparison, L cells are characterized by a polymorphic nucleus, low-level and dispersed DCF, and high OPD in the nucleus.

Neutrophils from four healthy donors were negatively isolated. The cells were stained with PE-CD66b, Hoechst, and DCF. Using BD FACSAria III, the cells were gated to CD66-positive cells and were displayed on a DCF vs. Hoechst scatter plot. The H and L cell clusters are shown in Figure 3A. Cells from the two clusters were isolated and pumped into two different tubes containing PFA as a fixative. After the cells were concentrated by a gentle spin (1000 RPM) and sealed in a glass chamber, the chamber was placed in the optical system and imaged by IPM. In addition, some of the cells were imaged by fluorescence and brightfield microscopy. The fluorescent stains are not expected to affect the OPD results, as the contribution of these molecules to the overall OPD value of the cell is negligible [22].

Fluorescent image analysis of the captured cells shows that H cells are represented by a round or banded nucleus (visualized by Hoechst binding to DNA) and granules of ROS (visualized by DCF fluorescent dye). In contrast, L cells are characterized by a mature, polymorphonuclear nucleus and a low level of ROS in the cytoplasm. After the H and L cells were sorted by the FACS machine, they were imaged using IPM. IPM results show that H cells display a higher OPD in the cytoplasm than in the nucleus, whereas L cells have a higher OPD in the nucleus than in the cytoplasm. Interestingly, granules that include ROS and are stained by DCF display localized high OPD levels (Figure 3B).

**Figure 4:**
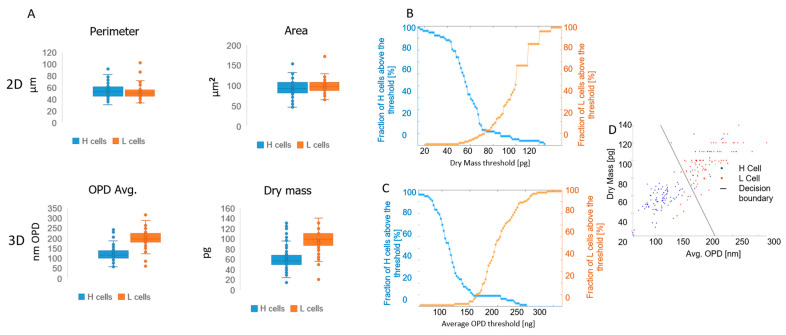
2D and 3D parameter statistics. Neutrophils were isolated from healthy BBDs and sorted by FACS into H and L cells. The cells were imaged by IPM and OPD maps of these cells were used to calculate 2D parameters (perimeter and area) and 3D parameters (average OPD and dry mass) in the two cell groups. Results show no statistically significant differences when comparing H and L cells in the 2D parameters; however, the 3D parameters are statistically significant (*p* < 0.0001) (**A**). In order to determine the optimal threshold to differentiate H and L cells, a CDF chart was used. The optimal threshold is the intersection point of the two curves: 73 pg (**B**) and 161 nm (**C**) for the dry mass and average OPD, respectively. A logistic linear regression model was used to combine the dry mass and the average OPD in a 2D model for the separation of H and L cells (**D**).

Overall, at this stage, 94 L cells and 77 H cells were captured. The cell images were converted to the label-free OPD maps and analyzed to extract different morphological parameters: two 2D parameters, cell area and cell perimeter, and two 3D parameters, average OPD and dry mass. The results from the 2D label-free parameters show no statistically significant differences between the two cell types: for cell area, the mean values are 100 vs. 98 μm^2^ for H and L cells, respectively, and for cell perimeter, the mean values are 54 vs. 51 μm for H and L cells, respectively. In contrast, 3D label-free parameters show statistically significant differences between the two cell types: mean average OPD values of 120 vs. 200 nm OPD and mean dry mass values of 59 vs. 96 μg for H and L cells, respectively, with *p* < 0.0001 in both cases (Figure 4A). 

In order to determine the threshold of dry mass that best distinguishes between the H and L cell populations, we computed the cumulative distribution function (CDF) of cell dry mass for both cell populations. The CDF of L cells and H cells was plotted (as shown in Figure 4B and Figure 4C, respectively) and the dry mass at which the two curves intersect was selected as the threshold that optimally separates these cell populations. This procedure was repeated for the average OPD, thereby yielding dry mass and average OPD thresholds of 73 μg and 161 μm, respectively, for distinguishing between the H and L cells. The accuracy of dry mass and OPD-based classifications using these thresholds was 88% and 90%, respectively.

In order to utilize both the dry mass and the average OPD, we used a 5-fold cross-validation logistic linear regression model for the separation of H and L cells. The model resulted in a 89% accuracy score (Figure 4D). 

### 3.3. Capturing Neutrophils from Healthy BBDs and MDS Patients

Blood samples from eight healthy BBDs and five MDS patients were analyzed. Neutrophils were isolated and the cells were stained by Hoechst and fixated by 1% PFA. Later, the cells were pumped into a microchannel at a rate of ~20 μL per hour. In case the cells were not visible as a result of sedimentation, the pumping speed was increased to 1000 μL/h for several minutes, after which the flow rate was returned to 20 μL per hour. The low flow rate was necessary to ensure that the cells flow on the bottom of the microchannel in the focal plane. The flowing cells were first visualized using fluorescent microscopy for the imaging of the nucleus (using Hoechst) in order to detect the presence of the cell, and upon detection, the cell was also imaged by IPM. This procedure was conducted in order to capture a single image per cell, rather than storing and processing large video files, thereby simplifying the data acquisition process. However, in the future, the cells may be imaged unstained and detected based on their phase values.

**Figure 5:**
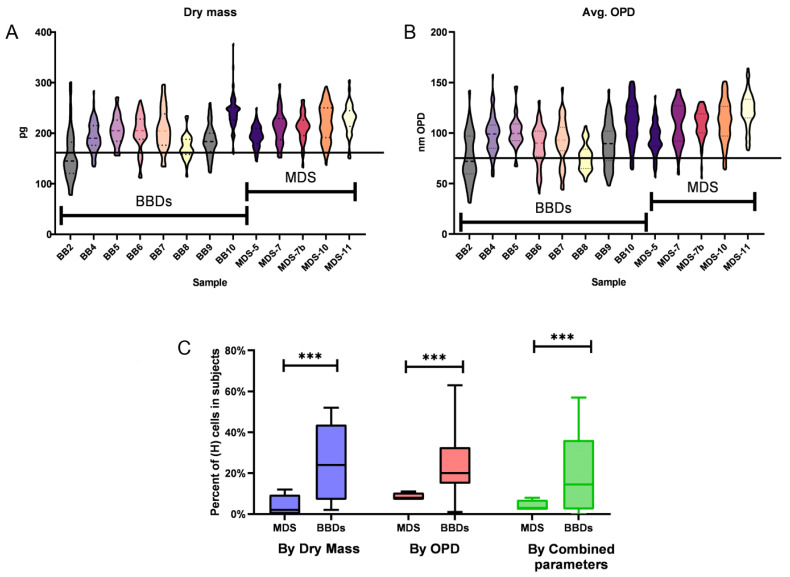
Violin plot charts of dry mass (**A**) and average OPD (**B**) of cells in MDS patients and BBDs. In both (**A**) and (**B**), the horizontal line represents the threshold between L and H cells. The percentage of H cells in MDS patients and BBDs presented in (**C**) are significantly different (*** represents *p* < 0.001).

The samples included 481 cells from MDS patients and 575 from BBDs. In order to exclude non-neutrophils from the analysis, cells that had a perimeter larger than the 95th percentile or smaller than the 5th percentile of sorted cells imaged were excluded. Thus, 438 neutrophils were analyzed for MDS patients and 384 for BBDs. Next, we used the threshold that was determined in Figure 3B in order to discriminate between H and L neutrophils by using the dry mass. The differences in dry mass and average OPD dispersion patterns (Figure 5A and Figure 5B, respectively) indicate the presence of H cell subpopulations mostly in BBDs, with these cells being characterized by dry mass or average OPD values below the threshold determined in Figure 4B.

The percentages of H cells obtained by the dry mass, the average OPD, and the combined parameters determined by logistic regression are displayed in Figure 4C. Thus, among the BBDs, 24%, 24%, and 20% of neutrophils are H cells, whereas in MDS patients, only 4%, 8.6%, and 4.2% are H cells, as calculated by the three methods mentioned above, respectively. The differences between the BBDs’ and MDS patients’ H cells ratio among neutrophils were found to be statistically significant (*p* < 0.001) using all cases.

As discussed in Section 3.1, MDS patient number 7 participated twice in the study, where in the second round (called MDS 7b), the blood sample was drawn after the patient underwent a blood transfusion in the same day. For this patient, the percentage of H cells increased from 3% to 28% using fluorescence flow cytometry. The latter measurement is typical of a healthy donor. In contrast, IPM showed 6% and 2% H cells, which still indicated an MDS patient in spite of the transfusion. Thus, IPM in this case is superior to fluorescence flow cytometry in its diagnostic value. 

## 4. Discussion

The diagnosis of MDS is a significant challenge in the current medical setting. In order to initiate the diagnostic process, the attending physician must first detect and interpret correctly the red flags that indicate the potential for MDS. The red flags include slowly progressing fatigue, bleeding infections, and normocytic or macrocytic anemia. Only after these red flags are sighted will the patient be referred for a bone marrow aspiration and biopsy in order to diagnose MDS conclusively. However, these red flags are very ambiguous and several years may pass until they are interpreted correctly by the attending physician.

The most common method for routine diagnosis of hematological pathologies is complete blood count (CBC), which measures cell volume based on a combination of electrical impedance and scatter analysis, as well as non-specific fluorescence labels to provide automated, low-cost, fast, and fairly reliable estimated five-part differential counting of WBCs: neutrophils, monocytes, eosinophils, basophils, and lymphocytes. However, for the more detailed morphological analyses of WBCs needed to diagnose MDS, a blood smear is needed, as this test can detect morphological abnormalities such as nuclear hyper-segmentation and cytoplasmic hypo-granularity [7]. In a blood smear, typically, manual analysis is performed under a light microscope, which is a labor-intensive method requiring skilled technicians and can take a long time [23]. This delayed diagnosis can result in the loss of therapeutic opportunities. For example, for higher-risk MDS patients, hematopoietic cell transplantation is a potential curative treatment, but it is available only for patients in good physical condition [9]. In addition, delayed diagnosis may delay the onset of palliative treatment that can improve quality of life [24]. Current translational research of MDS focuses on understanding the role of various genetic and epigenetic factors as prognostic or predictive markers to influence treatment. However, to date, these factors have little influence on treatment [25]. Our work presents a low-cost, low-skill, automated, and high-throughput approach for the raising of a red flag for MDS diagnosis. Employing our approach may improve the quality of life and survival of these patients.

In MDS patients, neutrophils may be deficient not only in number but also in anti-microbial functionality [26]. These functional impairments in neutrophils increase the risk of severe infections and infection-related deaths in MDS patients [5]. The functional deficiency of MDS neutrophils may be the result of hypo-granularity, which is a feature of MDS and other myeloid malignancies [27]. Our work demonstrates the decrease in granules in MDS patients as evidenced by the reduced presence of DCF-stained granules in neutrophils from MDS patients compared to those from BBDs.

Using flow cytometry, IPM, and fluorescence microscopy, we examined morphological differences between neutrophils derived from MDS patients and those obtained from healthy donors. When examining neutrophils from MDS patients and BBDs by using flow cytometry with stains for ROS (FDC) and DNA (Hoechst), a distinct pattern is observed. In BBDs, two cell clusters appear: one with higher Hoechst expression (H cells) and another with lower Hoechst expression (L cells). In contrast, MDS patients presented only L cells. The most likely reason for the difference between the two groups is different states of DNA decondensation, which affect the fluorescence with Hoechst staining [28,29]. Interestingly, H cells, found almost exclusively in the neutrophils of BBDs, are usually diverse in the quantity of ROS present in a single donor and the average ROS expression differs from sample to sample compared to the ROS in the L cells. This may suggest that the different BBDs presented different activation levels of the immune system during the time of donation. The fact that most MDS patients had no H cell cluster suggests a defective activation of the immune system. This correlates with the high rate of infections that MDS patients endure [5,30]. One possible explanation for the H cell population, found mostly in BBDs, is that these cells represent stimulated neutrophils that produce extracellular structures called neutrophil extracellular traps (NETs) [31]. During infection, neutrophils may swallow microorganisms through phagocytosis, following which the microorganisms are exposed to antimicrobial peptides, enzymes, and ROS. These components neutralize and kill the invading microorganisms, effectively preventing widespread infections [32]. In addition to phagocytosis and intracellular killing of pathogens, neutrophils are capable of neutralizing microorganisms by using NETs [33]. In this mechanism, the neutrophil secrets chromatin, including DNA and histones, to the extracellular area, thereby trapping pathogenic microorganisms. This process is dependent on the generation of ROS by NADPH oxidase [34]. In a recently published study, it was shown that neutrophils derived from MDS patients have markedly diminished NET-forming capacity relative to age-adjusted healthy controls; neutrophils from healthy controls present more chromatin decondensation and more ROS secretion of NETs in reaction to stimuli compared to neutrophils from MDS patients [35]. These results are in correlation with our morphological findings: the activation of the NET mechanism in BBDs but not in MDS patients is supported by the fact that H cells (found almost exclusively in BBDs) present higher Hoechst values, suggesting chromatin decondensation, and the presence of DCF staining is suggestive of ROS production. Decondensed DNA may also be the reason for the low OPD values of the nucleus in H cells. 

Our study has three main limitations. First, blood bank donors are not the ideal healthy control to MDS patients based on the differences in age. Second, a larger cohort of patients should be used in future research to strengthen our findings. Third, the present study includes only high-risk MDS cases. Initial results in low-risk MDS cases suggest that neutrophils in this condition do present H cells. A more comprehensive comparison between high-risk and low-risk MDS cells or anemia of unknown origin using IPM should be performed in future research. 

## 5. Conclusions

To date, automated CBC is the most commonly used tool for the diagnosis of hematological pathologies. For a more detailed morphological description of WBCs, manual analysis is needed, which is a labor-intensive method requiring skilled technicians and takes a long time to produce results. An IPM system integrated with a microfluidic mechanism has been shown to provide additional information on subtypes of WBCs that cannot be obtained by other automated methods and can potentially raise a red flag for MDS sooner. To our knowledge, this is the first study in which IPM has been used to distinguish between two WBCs of the same lineage in different developmental stages and thereby diagnose a medical condition, indicating that this technique may be applicable to numerous other pathologies. 

## Data Availability

The dataset is available on request from the authors.
